# Glycosaminoglycans in axon growth and regeneration: molecular mechanisms and therapeutic implications

**DOI:** 10.3389/fmolb.2026.1849064

**Published:** 2026-06-08

**Authors:** Kazuma Sakamoto, Yuko Nagai, Kenji Kadomatsu

**Affiliations:** 1 Department of Neuronal Information, Institute for Developmental Research, Aichi Developmental Disability Center, Kasugai, Aichi, Japan; 2 Institute for Glyco-core Research (iGCORE), Nagoya University, Nagoya, Aichi, Japan

**Keywords:** axon regeneration, chondroitin sulfate, glycosaminoglycan, heparan sulfate, protein tyrosine phosphatase sigma

## Abstract

Glycosaminoglycans (GAGs) are a structurally and chemically diverse family of sulfated polysaccharides that constitute a major component of the neural extracellular matrix and cell surface proteoglycans, where they exert pivotal regulatory functions in axon growth, guidance, synaptic organization, and regeneration. By forming highly specific and context-dependent interactions with axonal receptors, GAGs orchestrate the spatial patterning and temporal dynamics of signaling events after injury. Accumulating evidence indicates that the biological activities of GAGs are not dictated merely by their presence but are finely tuned by their sulfation codes, chain length, and domain organization. Recent mechanistic studies have revealed that distinct GAG species, particularly chondroitin sulfate (CS) and heparan sulfate (HS), exert opposing effects on axonal behavior through shared receptor systems. In the injured central nervous system (CNS), CS-rich extracellular matrices, prominently associated with reactive astrocytes and perineuronal nets, act as potent inhibitors of axon regeneration. These inhibitory effects are mediated through selective engagement of receptors such as protein tyrosine phosphatase sigma (PTPσ) leading to suppression of cytoskeletal dynamics and growth cone motility. In contrast, specific HS motifs promote axon elongation by inhibiting PTPσ. Based on these insights, therapeutic strategies targeting GAG biology have gained considerable attention. Approaches such as enzymatic digestion of inhibitory CS chains, development of synthetic or biomimetic GAGs, modulation of sulfation patterns, and gene editing of GAG-modifying enzymes have demonstrated encouraging efficacy in preclinical models of spinal cord injury, traumatic brain injury, and neurodegenerative disorders. Together, these findings indicate GAGs not only as passive structural components but as active, druggable regulators of axon growth and regeneration. This review integrates current advances in GAG structural biology, receptor interactions, and enzymatic regulation to provide a comprehensive framework for understanding how GAGs govern axonal behavior. We highlight unresolved questions and emerging opportunities for exploiting GAG-mediated mechanisms as actionable targets for next-generation neurorestorative therapies.

## 1 Introduction

Axon growth and regeneration are fundamental processes that shape the development, plasticity, and repair capacity of the nervous system (Silver and Miller, 2004). While neurons in the peripheral nervous system (PNS) retain a considerable ability to regenerate after injury, axon regeneration in the central nervous system (CNS) remains extremely limited (Huebner and Strittmatter, 2009). One of the earliest experimental demonstrations of this striking difference in regenerative capacity between the CNS and the PNS was clearly demonstrated by Aguayo and colleagues with “bridge” experiments (Benfey and Aguayo, 1982; David and Aguayo, 1981). In these seminal studies, a spinal cord injury model was established in which a segment of peripheral nerve, typically the sciatic nerve, was grafted to connect the rostral and caudal ends of the lesion. Remarkably, descending axons originating from the brain were able to extend into the grafted peripheral nerve and regenerate across the lesion site toward the distal segment. These observations indicated that the failure of axonal regeneration in the CNS is not primarily attributable to an intrinsic deficiency in neuronal growth capacity. Instead, they suggested that the extracellular environment of the CNS is fundamentally non-permissive for axon regrowth. Based on these findings, the authors proposed the existence of specific inhibitory components within the CNS milieu that restrict axonal regeneration but are absent from the PNS. This interpretation is further supported by the behavior of neurons that project axons to both the CNS and PNS, such as dorsal root ganglion (DRG) neurons. Following injury, these neurons typically regenerate their peripheral axons while their central branches fail to regrow, highlighting the critical role of environmental cues in determining regenerative outcomes.

This regenerative failure in the CNS is largely attributed to the inhibitory extracellular environment that emerges following injury, including the formation of glial scars and the accumulation of inhibitory extracellular matrix (ECM) molecules (Silver and Miller, 2004; Yiu and He, 2006; Kadomatsu and Sakamoto, 2014a; Kadomatsu and Sakamoto, 2014b). Among these components, proteoglycans bearing glycosaminoglycans (GAGs) chains have emerged as key regulators of axonal behavior (Kadomatsu and Sakamoto, 2014a; Kadomatsu and Sakamoto, 2014b).

GAGs are linear and sulfated polysaccharides that decorate a variety of cell-surface and extracellular matrix proteoglycans (Sugahara and Mikami, 2007). In the nervous system, GAGs are abundant within the neural ECM and play important roles in neuronal development, synaptic organization, and structural plasticity (Sakamoto et al., 2021a). Major GAG classes present in neural tissues include chondroitin sulfate (CS), heparan sulfate (HS), keratan sulfate (KS), and dermatan sulfate (DS), each characterized by distinct disaccharide compositions, sulfation patterns, and structural domains (Sugahara and Mikami, 2007; Margolis and Margolis, 1993). These structural features create a high degree of molecular diversity, enabling GAGs to engage in selective interactions with a wide range of signaling molecules, growth factors, and cell-surface receptors.

Increasing evidence indicates that GAGs function not merely as passive structural components of the extracellular matrix but as active regulators of cellular signaling. In particular, the sulfation pattern and domain organization of GAG chains are now recognized as critical determinants of their biological activity. This concept, so-called “GAG sulfation code,” proposes that specific combinations of sulfation motifs encode distinct biological functions by controlling ligand binding and receptor activation. Within the nervous system, such structural codes may determine whether the extracellular environment promotes or restricts axon growth. The role of GAGs in axon regeneration has been most extensively studied in the context of CNS injury and other CNS pathologies. CS proteoglycans (CSPGs), which accumulate within glial scars and perineuronal nets, are widely recognized as potent inhibitors of axonal regeneration (Snow et al., 1990; Snow et al., 1991; McKeon et al., 1995; Smith-Thomas et al., 1994). Mechanistically, CS chains interact with specific neuronal receptors such as protein tyrosine phosphatase sigma (PTPσ) (Shen et al., 2009; Coles et al., 2011), leukocyte common antigen–related phosphatase (LAR) (Fisher et al., 2011), and members of the Nogo receptor family (Dickendesher et al., 2012), triggering intracellular signaling cascades that suppress cytoskeletal remodeling and growth cone motility. In contrast, HS has been shown to facilitate axon growth and guidance in multiple contexts, in part through modulation of receptor signaling pathways and interactions with growth factors (Inatani et al., 2003; Wang and Denburg, 1992). KS represents another class of GAGs that has been implicated in the inhibition of axonal regeneration (Cole et al., 1991). KS is enriched in several extracellular matrix proteoglycans present in the central nervous system and has been reported to accumulate in lesion environments following neural injury (Snow et al., 1990; Smith-Thomas et al., 1994; Cole et al., 1991; Jones and Tuszynski, 2002; Krautstrunk et al., 2002). Similar to CSPGs, KS-containing proteoglycans (KSPGs) are thought to contribute to the formation of a growth-inhibitory extracellular milieu that restricts axonal extension (Kadomatsu and Sakamoto, 2014a; Kadomatsu and Sakamoto, 2014b; Imagama et al., 2011; Ito et al., 2010). However, in contrast to CSPGs, the specific receptors that mediate KS-dependent signaling in neurons have not yet been clearly identified. The molecular mechanisms by which KS transduces inhibitory signals to the intracellular machinery therefore remain largely unresolved and represent an important area for future investigation. In contrast, DS has been shown to exert pro-regenerative effects on axonal growth (Hikino et al., 2003). Previous studies have demonstrated that DS can promote neurite extension, at least in part through signaling mediated by the receptor tyrosine kinase anaplastic lymphoma kinase (ALK) (Machino et al., 2021). Activation of this pathway appears to stimulate intracellular signaling cascades that support cytoskeletal dynamics and neurite elongation. These observations highlight the functional diversity of GAGs in the nervous system, where distinct sulfation patterns and structural motifs can differentially regulate neuronal growth either by inhibiting or promoting axonal regeneration.

Recent advances in glycobiology, structural analysis, and neurobiology have significantly expanded our understanding of how GAGs regulate axonal signaling. Studies have begun to reveal how specific sulfation motifs, chain lengths, and domain organizations determine receptor binding and downstream signaling outcomes. At the same time, growing interest has emerged in targeting GAG-mediated pathways for therapeutic intervention. Strategies including enzymatic degradation of inhibitory CS chains, manipulation of sulfation patterns, development of synthetic or biomimetic GAG molecules, genetic modulation of GAG biosynthetic enzymes, and direct targeting of GAG receptors have shown promising results in experimental models of CNS injury and neurodegenerative diseases.

Despite these advances, many fundamental questions remain unresolved. The precise structural features that govern receptor specificity, the mechanisms by which different GAG species generate opposing signaling outputs, and the broader principles that define the functional “GAG code” in neural tissues are still incompletely understood. Addressing these questions is essential for translating insights from glycobiology into effective neurorestorative therapies.

In this review, we summarize current knowledge regarding the structural diversity of GAGs, their interactions with neuronal receptors, and their roles in regulating axon growth and regeneration. Emerging evidence suggests that distinct GAG species can exert opposing regulatory effects on axonal signaling through shared receptor systems. In particular, CS and HS have been proposed to differentially regulate PTPσ signaling, thereby influencing growth cone behavior and autophagic flux (Sakamoto et al., 2019; Tran et al., 2020). Understanding this molecular logic may provide a conceptual framework for the “GAG code” that governs axonal responses in the injured CNS. We further discuss emerging therapeutic strategies targeting GAG-mediated signaling and highlight key challenges and future directions in this rapidly evolving field.

## 2 Structural diversity of GAGs

GAGs are a class of linear polysaccharides composed of repeating disaccharide units that exhibit remarkable structural diversity and functional versatility (Afratis et al., 2012; Karamanos and Tzanakakis, 2012; Ricard-Blum et al., 2024). These macromolecules are typically covalently attached to core proteins to form proteoglycans, which represent major structural and signaling components of the extracellular matrix (ECM) and cell surface in neural tissues. Proteoglycans bearing GAG chains are widely distributed throughout the CNS, where they contribute not only to the physical architecture of the extracellular environment but also to the regulation of cellular communication and molecular signaling. Through their extended and highly hydrated polymeric structures, GAGs provide a scaffold that organizes extracellular molecules, modulates receptor–ligand interactions, and influences cell adhesion, migration, and differentiation.

The structural complexity of GAGs arises from multiple levels of molecular variation. These include differences in monosaccharide composition, sulfation patterns, uronic acid epimerization, chain length, and domain organization along the polysaccharide chain. In addition, the distribution of sulfated and non-sulfated regions along a single GAG chain can create distinct microdomains that selectively interact with a variety of proteins. This structural heterogeneity allows GAGs to bind to numerous signaling molecules such as growth factors, cytokines, chemokines, and axon guidance cues. As a result, GAGs function as key modulators of extracellular signaling networks that regulate neuronal development, synaptic plasticity, and responses to neural injury. In the context of the nervous system, such structural diversity enables GAGs to participate in finely tuned molecular interactions that influence processes including axon guidance, synapse formation, neuroinflammation, and regeneration.

**FIGURE 1:**
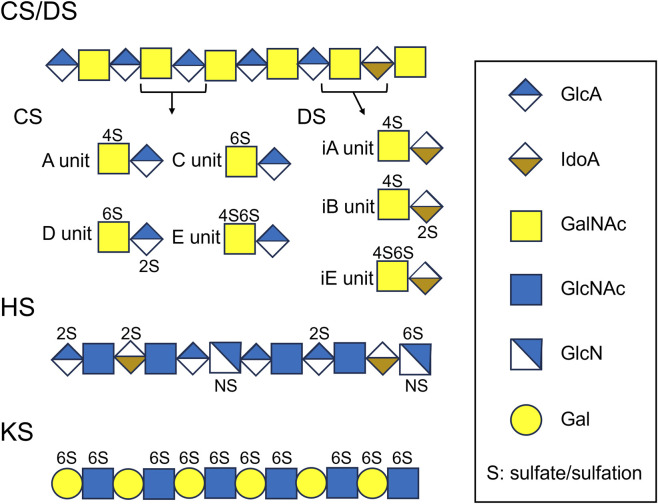
Structural diversity of major sulfated GAGs. Schematic representation of the basic disaccharide structures and characteristic sulfation patterns of major GAGs. Chondroitin sulfate (CS) and dermatan sulfate (DS) consist of repeating disaccharides of glucuronic acid (GlcA) or iduronic acid (IdoA) linked to *N*-acetylgalactosamine (GalNAc), with different sulfation motifs generating distinct subtypes including CS-A (4-*O*-sulfation), CS-C (6-*O*-sulfation), CS-D (2,6-*O*-sulfation), and CS-E (4,6-*O*-sulfation). Heparan sulfate (HS) is composed of alternating uronic acid (GlcA or IdoA) and glucosamine residues, which undergo *N*-sulfation and *O*-sulfation at multiple positions, producing highly heterogeneous sulfation domains. Keratan sulfate (KS) contains repeating galactose (Gal) and *N*-acetylglucosamine (GlcNAc) units, typically modified by 6-O-sulfation. “S” represents sulftate/sulfation. Symbols follow the SNFG notation.

The major GAG species present in the CNS include chondroitin sulfate (CS), heparan sulfate (HS), keratan sulfate (KS), and dermatan sulfate (DS) (Margolis and Margolis, 1993) (Figure 1). Although each of these GAGs is composed of repeating disaccharide units, they differ in their specific monosaccharide constituents as well as in their sulfation and epimerization patterns. These structural differences confer distinct biochemical properties and biological functions on each GAG family. For example, CS consists of repeating units of *N*-acetylgalactosamine (GalNAc) and glucuronic acid (GlcA), which can be variably sulfated at different positions to generate diverse CS subtypes. In contrast, HS is composed of repeating units of *N*-acetylglucosamine (GlcNAc) and either glucuronic acid or iduronic acid. HS chains undergo extensive post-polymerization modifications, including *N*-sulfation, *O*-sulfation, and epimerization, resulting in highly heterogeneous sulfation patterns that are critical for binding growth factors and morphogens.

DS is structurally related to CS but differs in that a substantial proportion of its glucuronic acid residues are converted to iduronic acid through C5 epimerization. This epimerization alters the conformational flexibility of the polysaccharide chain and can significantly influence its protein-binding properties (Mizumoto and Yamada, 2022). KS, in contrast to the uronic acid–containing GAGs, is composed of repeating disaccharide units of GlcNAc and galactose. KS chains are typically sulfated at the C6 position of these sugars and lack uronic acid residues, which gives them unique physicochemical properties compared with other GAG families (Funderburgh, 2000; Funderburgh, 2002).

A defining feature of GAG structural diversity is the extensive modification of the polysaccharide chains through sulfation. Sulfate groups can be attached at multiple positions on the sugar residues, generating a variety of sulfation motifs. In CS chains, sulfation typically occurs at the 4- or 6-position of GalNAc, producing the well-known CS-A and CS-C motifs, while additional sulfation can generate more complex structures such as CS-D and CS-E. In HS, sulfation occurs at several positions, including *N*-sulfation of glucosamine and sulfation at the 2-, 3-, and 6-positions, resulting in an exceptionally large repertoire of possible sulfation patterns. As for KS, the 6-sulfation of GlcNAc is a key structural modification required for the biosynthesis of KS chains (Zhang et al., 2006). In addition, galactose residues within the KS backbone can also be sulfated at the C6 position, contributing to the structural diversity of KS. KS proteoglycans (KSPGs) are linked to their core proteins through several distinct glycan linkages, including *N*-linked, *O*-linked, and *O*-mannose-linked glycosylation. These modifications are introduced by specific sulfotransferases and epimerase during biosynthesis in the Golgi apparatus, leading to the generation of highly heterogeneous GAG chains.

Beyond sulfation, additional layers of structural diversity arise from variations in chain length and domain organization. GAG chains are not uniform along their entire length but instead contain regions enriched in specific modification patterns. In HS, for example, highly sulfated domains are interspersed with less-modified regions, forming a domain architecture that influences ligand recognition and receptor binding (Esko and Selleck, 2002; Murphy et al., 2004). Similar domain structures have also been reported in CS chains (Sugahara et al., 2003), where clusters of particular sulfation motifs can create functional binding sites for specific proteins. This domain-based organization allows GAG chains to act as molecular platforms that selectively recruit signaling molecules.

These structural variations collectively give rise to the concept that GAGs encode biological information through specific combinations of sulfation motifs and structural domains (Esko and Selleck, 2002; Sarrazin et al., 2011). Rather than functioning solely as passive scaffolds, GAG chains can regulate cellular signaling by controlling the availability, localization, and activity of growth factors and receptors. In the nervous system, such structural codes are thought to play critical roles in determining whether the extracellular environment promotes or restricts axon regeneration.

Understanding the structural diversity of GAGs is therefore essential for elucidating how these molecules regulate neuronal behavior. Advances in analytical technologies, including mass spectrometry–based glycomics and glycoproteomics, have begun to reveal the complexity of GAG structures in neural tissues. These approaches are providing new insights into how specific structural motifs contribute to receptor interactions and downstream signaling pathways. In the following sections, we discuss how distinct GAG species and their structural features influence axonal signaling and regeneration within the CNS.

## 3 GAG receptors in axonal signaling

**FIGURE 2:**
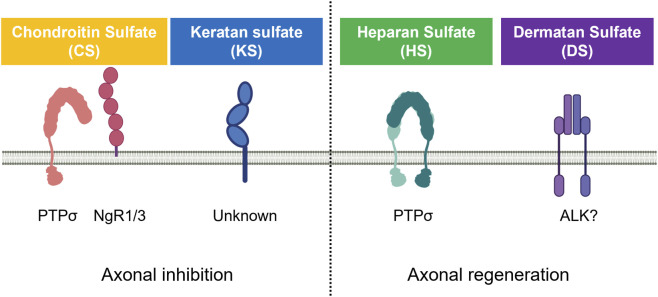
Differential roles of GAGs in axonal growth regulation. Schematic illustration of the distinct effects of GAGs on axonal growth through receptor-mediated signaling pathways. CS inhibits axonal extension primarily through receptors such as PTPσ and NgR1/3. KS has also been implicated in axonal inhibition, although its specific receptor remains unclear. In contrast, HS promotes axonal regeneration, partly through inactivation of PTPσ that counteract CSPG-mediated inhibition. DS has been reported to enhance axonal growth, potentially via signaling pathways involving receptors such as anaplastic lymphoma kinase (ALK).

A growing body of evidence indicates that the biological effects of GAGs on axonal behavior are mediated through specific interactions with neuronal cell-surface receptors (Figure 2). Rather than acting solely as passive structural components of the ECM, GAG chains are now widely recognized as bioactive molecules that function as instructive regulators of cellular signaling. Through their diverse sulfation patterns and domain organizations, GAGs are capable of selectively interacting with a variety of proteins, including growth factors, guidance cues, and cell-surface receptors. These interactions enable GAGs to influence multiple aspects of neuronal physiology, including neurite outgrowth, axon guidance, synaptic plasticity, and regenerative responses following injury.

In the injured CNS, the ECM undergoes extensive remodeling, leading to the accumulation of GAG-rich proteoglycans within the glial scar (Silver and Miller, 2004; Yiu and He, 2006). This molecular environment provides not only a structural barrier but also a potent biochemical signaling platform that modulates neuronal behavior. Several receptor systems have been identified that selectively recognize sulfated GAG chains and transduce inhibitory or modulatory signals within neurons. These receptors detect specific sulfation motifs within GAG chains and convert extracellular molecular cues into intracellular signaling cascades that regulate cytoskeletal organization, growth cone dynamics, and axonal extension.

Among the receptors that mediate these interactions, receptor-type protein tyrosine phosphatases, including protein tyrosine phosphatase sigma (PTPσ) and leukocyte common antigen–related phosphatase (LAR), have emerged as key components of the GAG signaling machinery (Shen et al., 2009; Coles et al., 2011; Fisher et al., 2011; Fox and Zinn, 2005; Aricescu et al., 2002). In addition, members of the Nogo receptor family have been shown to bind sulfated GAGs and contribute to the transduction of inhibitory signals that limit axonal regeneration in the CNS (Dickendesher et al., 2012). Together, these receptor systems form a complex molecular interface through which extracellular GAGs regulate neuronal responses to injury. Understanding how these receptors recognize distinct GAG structures and activate downstream signaling pathways is therefore essential for elucidating the mechanisms that control axonal growth and regeneration in the damaged CNS.

### 3.1 PTPσ

PTPσ is one of the best-characterized receptors that mediate the inhibitory effects of chondroitin sulfate proteoglycans (CSPGs) on axon growth (Shen et al., 2009; Coles et al., 2011; Sakamoto et al., 2019). PTPσ belongs to the leukocyte common antigen-related (LAR) family of receptor-type protein tyrosine phosphatases and contains extracellular immunoglobulin-like domains that enable direct binding to sulfated GAGs chains (Tonks, 2006). Biochemical and structural studies have demonstrated that CS chains bind to the extracellular domain of PTPσ and induce receptor monomerization, which in turn enhances intracellular phosphatase activity and downstream signaling pathways (Shen et al., 2009; Coles et al., 2011; Sakamoto et al., 2019) (Figure 2).

Activation of PTPσ signaling has been shown to suppress axon extension by regulating cytoskeletal dynamics and growth cone behavior. In particular, engagement of CSPGs with PTPσ can trigger signaling cascades that influence actin stability and autophagy flux, ultimately leading to formation of dystrophic endbulb and reduced axon elongation (Sakamoto et al., 2019). Genetic deletion or pharmacological inhibition of PTPσ has been reported to enhance axon regeneration in several experimental models of axonal injury (Gardner et al., 2015; Lang et al., 2015; Tran et al., 2018; Warren et al., 2018), highlighting the central role of this receptor in mediating the inhibitory effects of the glial scar environment.

Interestingly, recent studies have revealed that HS can interact with PTPσ in a manner distinct from CS (Aricescu et al., 2002; Sakamoto et al., 2019). While CS binding generally promotes inhibitory signaling, HS chains have been suggested to promote PTPσ clustering, thereby attenuating inhibitory signaling and promoting axonal growth (Figure 2). These observations suggest that different GAG species may compete for or differentially modulate the same receptor, providing a molecular mechanism through which extracellular matrix composition influences neuronal responses to injury.

### 3.2 LAR

In addition to PTPσ, other members of the LAR family of receptor-type protein tyrosine phosphatases play important roles in GAG-mediated signaling (Fisher et al., 2011; Fox and Zinn, 2005). LAR itself shares structural similarity with PTPσ and contains extracellular domains capable of interacting with sulfated GAG chains. Like PTPσ, LAR has been shown to bind CSPGs and to mediate inhibitory signaling that restricts axon growth.

Functional studies indicate that LAR contributes to the inhibitory effects of CSPGs on neuronal regeneration. Disruption of LAR signaling through genetic manipulation can partially alleviate CSPG-induced inhibition of axon growth (Fisher et al., 2011). These findings suggest that multiple LAR family receptors cooperate to transduce inhibitory signals triggered by GAG-rich extracellular matrices.

Beyond their inhibitory roles, LAR family phosphatases are also implicated in broader aspects of neuronal development, including synapse formation and axon guidance (Sakamoto et al., 2021a). Their ability to interact with diverse extracellular ligands, including both proteoglycans and cell adhesion molecules, positions these receptors as central integrators of extracellular cues. Consequently, the balance between different GAG ligands and receptor interactions may critically influence neuronal plasticity and regenerative responses.

### 3.3 Nogo receptor family

NgR1 and NgR3, members of the Nogo receptor (NgR) family, represent another class of receptors involved in the recognition of inhibitory extracellular cues in the CNS (Dickendesher et al., 2012). Although these receptors were originally identified as binding partners for myelin-associated inhibitors such as Nogo-A, subsequent studies have demonstrated that NgRs can also interact with certain GAGs, especially CS-D and CS-E.

NgR-mediated signaling converges on intracellular pathways that regulate cytoskeletal organization and growth cone dynamics. Activation of NgR complexes typically leads to RhoA signaling and inhibition of axonal extension. It is possible that CSPGs may cooperate with myelin-associated inhibitors to reinforce inhibitory signaling through NgR-containing receptor complexes, thereby contributing to the non-permissive environment that develops after CNS injury.

The interplay between NgRs and other GAG-binding receptors, including PTPσ and LAR, remains an active area of investigation. It is increasingly recognized that axonal responses to extracellular inhibitors are not mediated by a single receptor pathway but rather arise from the coordinated activity of multiple receptor systems that integrate diverse environmental signals.

### 3.4 Others

KS has also been shown to strongly inhibit axonal regeneration *in vitro* (Cole et al., 1991). As described above, 6-sulfation of GlcNAc is a critical modification required for KS chain elongation in the CNS (Zhang et al., 2006). Consistent with this, genetic disruption of GlcNAc6ST-1, the enzyme responsible for this sulfation reaction, leads to a loss of KS expression. In a brain stab injury model, mice lacking KS exhibited significantly enhanced neurite outgrowth around the lesion site compared with wild-type animals (Zhang et al., 2006). Similarly, in a mouse model of spinal cord injury, KS-deficient mice showed improved axonal regeneration and enhanced hindlimb motor function following injury (Ito et al., 2010). Furthermore, intrathecal administration of keratanase II, a bacterial enzyme that specifically degrades KS, promoted axonal regeneration and functional recovery in rats with thoracic spinal cord injury (Imagama et al., 2011). Notably, the magnitude of recovery was comparable to that observed with treatment using chondroitinase ABC (ChABC), a well-established enzyme that degrades CS. Although these findings collectively support the idea that KS acts as an inhibitory component of the ECM that restricts axonal regeneration after CNS injury, a specific receptor that mediates KS-dependent inhibitory signaling has not yet been clearly identified.

In contrast, DS has been reported to promote neurite outgrowth (Hikino et al., 2003), and ALK has been proposed as a candidate receptor mediating this effect (Machino et al., 2021). However, the molecular mechanisms underlying DS-dependent signaling remain incompletely understood. In addition, ALK has also been reported to interact with HS, raising the possibility that multiple GAGs may modulate ALK-dependent signaling pathways in the regulation of neurite growth (Murray et al., 2015).

### 3.5 Integration of GAG-dependent signaling pathways

Together, these receptor systems illustrate how GAG chains act as molecular regulators of axonal signaling. The structural diversity of GAGs—including variations in sulfation motifs and domain organization—likely determines their affinity for specific receptors and the signaling outcomes that follow. Consequently, changes in extracellular matrix composition after injury may alter the balance of receptor activation and thereby influence the regenerative capacity of neurons.

Understanding the molecular mechanisms that govern GAG–receptor interactions is therefore critical for developing therapeutic strategies aimed at promoting axon regeneration. Targeting these signaling pathways, either by disrupting inhibitory receptor engagement or by enhancing permissive GAG interactions, represents a promising avenue for restoring neural connectivity after CNS injury.

## 4 Opposing functions of chondroitin sulfate and heparan sulfate in axonal regulation

A key unresolved question is how structurally related sulfated glycans can produce opposite biological outcomes through the same receptor. In particular, among the diverse GAG species present in the nervous system, CS and HS have emerged as key regulators of axonal behavior, especially through PTPσ (Shen et al., 2009; Coles et al., 2011). Although both molecules belong to the same family of sulfated polysaccharides and share similar structural principles, accumulating evidence indicates that they exert markedly different—and often opposing—effects on axon growth and regeneration. Understanding how these structurally related glycans generate contrasting biological outcomes is essential for elucidating the regulatory logic of the neural extracellular matrix. One proposed model suggests that CS promote PTPσ monomerization and activation, whereas HS may induce PTPσ clustering that suppresses phosphatase signaling (Shen et al., 2009; Coles et al., 2011).

In our earlier work, we explored how CS and HS differentially regulate PTPσ and consequently influence axonal responses after injury (Sakamoto et al., 2021a; Sakamoto et al., 2019; Sakamoto et al., 2021b). The structural heterogeneity of GAG chains, particularly in their sulfation patterns, presents a major challenge in identifying the specific motifs responsible for receptor binding. To overcome this limitation, we generated a panel of chemically defined CS and HS oligosaccharides that differed in chain length and sulfation configuration. Using surface plasmon resonance assays, we first characterized the interactions between PTPσ and several CS isoforms. Among the variants examined, including CS-A, CS-C, CS-D, and CS-E, the CS-E species displayed the highest binding affinity toward PTPσ. CS-E has the GalNAc residue carrying both C4 and C6 sulfate groups, suggesting that this dual sulfation pattern plays a critical role in receptor recognition.

**FIGURE 3:**
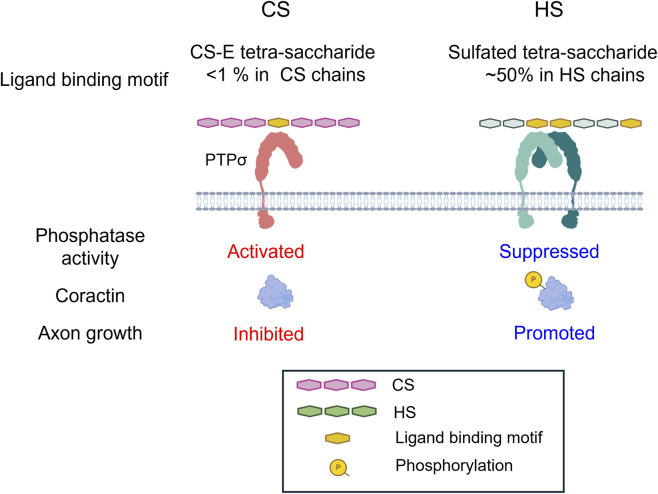
Differential regulation of PTPσ signaling by CS and HS. Schematic model illustrating how CS and HS oppositely regulate PTPσ organization and downstream signaling during axonal growth. CS chains containing rare CS-E sulfation motifs induce PTPσ monomerization, resulting in activation of phosphatase activity. Activated PTPσ promotes cortactin dephosphorylation, ultimately leading to inhibition of axon growth and regenerative failure. In contrast, sulfated HS motifs, which are more frequently distributed along HS chains, promote PTPσ clustering and suppress phosphatase activity. This clustered state preserves cortactin phosphorylation, thereby supporting growth cone extension and axon regeneration.

To further delineate the minimal structural motif required for PTPσ binding, we analyzed CS-E oligosaccharides of varying lengths. These experiments demonstrated that a tetrasaccharide represents the smallest structural unit capable of interacting with the receptor. Interestingly, the CS-E motif is relatively uncommon and constitutes only a minor proportion of total CS chains within the injured rodent CNS (Properzi et al., 2005). As a result, a CS-E tetrasaccharide is predicted to occur at most once within an individual CS chain (Figure 3). This structural arrangement suggests that CS chains may be configured in a manner that promotes PTPσ monomerization and receptor activation (Figure 2). In contrast, analyses of HS oligosaccharides revealed that HS structures containing one or more sulfate groups were sufficient to bind PTPσ. Because such sulfated HS motifs account for nearly half of the HS population in the injured CNS (Properzi et al., 2008), HS chains may instead facilitate receptor multimerization, thereby functionally suppressing PTPσ signaling (Figures 2, 3). Consistent with this idea, cell culture experiments using synthetic CS and HS oligosaccharides supported a model in which differences in sulfation pattern and domain frequency determine the opposing effects of CS and HS on PTPσ regulation. Although the precise molecular basis for these interactions remains to be fully elucidated, our findings indicate that the distribution and frequency of receptor-binding domains within GAG chains play a key role in modulating PTPσ activity.

To gain further insight into the cellular consequences of CS-induced PTPσ activation, we next investigated the morphology of dystrophic endbulbs, that form along CS gradients *in vitro* using electron microscopy. Dystrophic endbulbs are swollen axon terminals that form after CNS injury when regenerating axons fail to progress and become stalled at inhibitory environments such as the glial scar (Tom et al., 2004). These observations revealed a striking accumulation of autophagosomes within dystrophic endbulbs. Immunostaining with LC3, a widely used marker of autophagosomes, confirmed this ultrastructural finding. Notably, a similar enrichment of autophagosomes was also detected at the distal ends of severed corticospinal tract axons in a mouse model of spinal cord injury.

Autophagy is a conserved intracellular degradation system that mediates the turnover of cytoplasmic components and organelles (Mizushima and Komatsu, 2011). The process begins with the formation of a cup-shaped isolation membrane, termed the phagophore, which frequently originates at membrane contact sites between the endoplasmic reticulum and mitochondria. This structure expands to engulf portions of cytoplasm and organelles, ultimately forming a double-membrane vesicle known as the autophagosome. The autophagosome subsequently fuses with lysosomes to generate an autolysosome, in which the sequestered material is degraded by lysosomal hydrolases. In neurons, autophagosome biogenesis occurs predominantly at distal axonal regions. Newly formed autophagosomes are then transported retrogradely along microtubules toward the soma while undergoing progressive fusion with lysosomes during their transport (Maday et al., 2012; Maday and Holzbaur, 2014; Maday et al., 2014). Because extending axons require continuous remodeling and turnover of cellular components—including cytoskeletal elements, mitochondria, and membrane compartments—autophagy is considered a critical mechanism for maintaining homeostasis at growing axonal tips.

**FIGURE 4:**
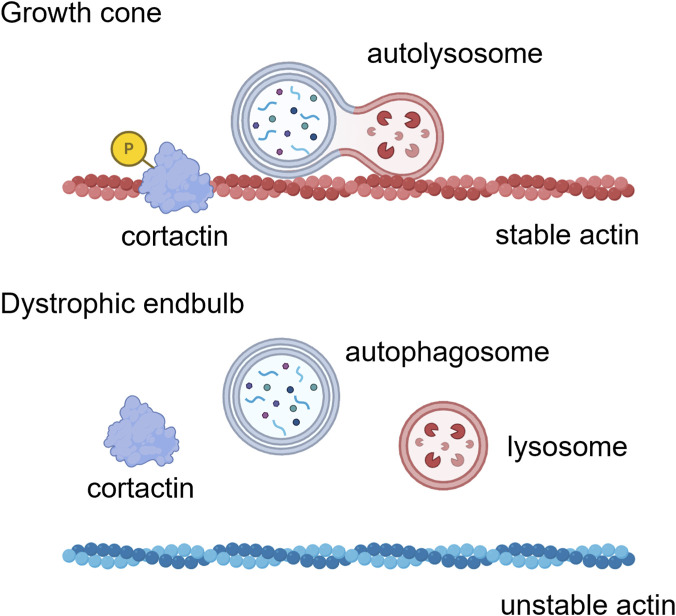
CSPG-mediated inhibition of axonal regeneration. Schematic illustration of the mechanisms by which CSPGs inhibit axonal regeneration after CNS injury. (upper) In the absence of CS, the inhibitory signaling mediated by PTPσ is attenuated. As a result, phosphorylation of cortactin is maintained, leading to stabilization of the actin cytoskeleton. This stabilization facilitates the trafficking and fusion of autophagosomes with lysosomes, thereby promoting efficient autophagic flux and supporting axonal growth. (lower) After injury, CS activates PTPσ, leading to dephosphorylation of cortactin. The loss of cortactin phosphorylation destabilizes actin filaments and disrupts cytoskeletal organization. Consequently, the trafficking and fusion of autophagosomes with lysosomes are impaired, resulting in defective autophagic flux. This autophagy dysfunction contributes to the accumulation of vesicular structures at injured axon tips and ultimately leads to the formation of dystrophic endbulbs.

An increase in autophagosome number may reflect either enhanced induction of autophagy or impairment of autophagic flux, particularly at the stage of autophagosome–lysosome fusion. To discriminate between these possibilities, we performed immunocytochemical analyses using tandem fluorescent LC3 (Kimura et al., 2007), a reporter that allows differential visualization of autophagosomes and autolysosomes. These analyses revealed that autophagic flux was markedly disrupted in dystrophic endbulbs. Consistent with this interpretation, RNA interference–mediated depletion of Syntaxin 17, VAMP8, or SNAP29—SNARE proteins essential for autophagosome–lysosome fusion (Itakura et al., 2012)—converted normal growth cones into dystrophic endbulb–like structures and markedly suppressed axonal extension in cultured mouse dorsal root ganglion neurons. Similar phenotypes were also observed following pharmacological inhibition of autophagosome–lysosome fusion using chloroquine or bafilomycin A1. Taken together, these findings indicate that disruption of autophagic flux is both necessary and sufficient to drive the formation of dystrophic endbulbs (Figure 4).

To further elucidate how PTPσ signaling is mechanistically linked to autophagy, we next sought to identify downstream substrates of PTPσ involved in regulating the fusion step between autophagosomes and lysosomes. Particular attention was directed to cortactin, an actin-binding protein that plays a critical role in facilitating this fusion process (Hasegawa et al., 2016). Cortactin possesses multiple tyrosine phosphorylation sites, among which phosphorylation at tyrosine residues 421 and 466 is required for stabilizing actin filaments (Hasegawa et al., 2016). A fraction of tyrosine-phosphorylated cortactin localizes specifically to lysosomal membranes via protein–lipid interactions, where it promotes the formation of stabilized actin networks that support autophagosome–lysosome fusion (Figure 4).

In primary dorsal root ganglion neurons cultured on a CSPG gradient, we observed a marked reduction in cortactin phosphorylation at tyrosine 421 within dystrophic endbulbs compared with normal growth cones. Furthermore, depletion of cortactin induced the formation of dystrophic endbulb–like structures resembling those produced by CS treatment. These observations suggest a model in which CS engagement of PTPσ activates the phosphatase, leading to cortactin dephosphorylation. Loss of cortactin phosphorylation disrupts the completion of autophagic flux, ultimately converting dynamic growth cones into inactive dystrophic endbulbs.

The opposing functions of CS and HS have led to the proposal that GAGs operate through a molecular “code” that regulates neuronal behavior. According to this concept, specific combinations of sulfation motifs, domain structures, and chain lengths generate distinct biochemical signals that influence receptor activation and downstream signaling pathways.

Within the injured CNS, changes in the relative abundance and structural composition of different GAG species may therefore determine whether the extracellular environment becomes permissive or inhibitory for axon regeneration. For example, the accumulation of CS-rich matrices within glial scars creates a strongly inhibitory microenvironment, whereas HS-rich regions may support growth-promoting signaling pathways.

Deciphering this putative GAG code represents a major challenge in modern glycobiology and neurobiology. Advances in analytical technologies, including high-resolution mass spectrometry and glycomic profiling, are beginning to provide new insights into the structural organization of GAG chains in neural tissues. A deeper understanding of how specific GAG motifs regulate receptor signaling will be critical for developing therapeutic strategies aimed at manipulating extracellular matrix signals to promote neural repair.

## 5 Therapeutic targeting of GAG signaling

The recognition that GAGs function as active regulators of axonal signaling has stimulated considerable interest in targeting these molecules for therapeutic intervention. Because GAG-mediated pathways play central roles in shaping the extracellular environment after central nervous system (CNS) injury, strategies aimed at modifying GAG structures or disrupting their interactions with neuronal receptors have emerged as promising approaches to promote neural repair. A variety of experimental strategies have been explored, including enzymatic degradation of inhibitory GAG chains, manipulation of sulfation patterns, development of synthetic or biomimetic glycans, and genetic modulation of GAG biosynthetic enzymes (Table 1).

**TABLE 1:** Therapeutic strategies targeting GAG signaling to promote axon regeneration.

Strategy	Methods	Aim/Results	References
GAG degradation	ChABC	Degradation of CS	Bradbury et al. (2002), Moon et al. (2001), Massey et al. (2006), Gogolla et al. (2009), Pizzorusso et al. (2002), García-Alías et al. (2009)
	Keratanase II	Degradation of KS	Imagama et al. (2011)
GAG mimetics	Enoxaparin	Antagonize PTP	Ito et al. (2021)
	Cellulose sulfate	Permissive scaffold	Menezes et al. (2019)
Sulfation modification	CS-E antibody	CS-E blockade	Brown et al. (2012)
	protamine	CS-E blockade	Ozaki et al. (2025)
Gene targeting	GlcNAc6ST1 KD	Reduced KS	Ito et al. (2010), Zhang et al. (2006)
	CSGalNAcT1 KD	Reduced CS, increased HS	Takeuchi et al. (2013)
	Chsy1 KD	Reduced CS	Liu et al., 2023
Receptor targeting	Wedge peptide (NVG-291)	Inactivate PTP	Gardner et al. (2015), Lang et al. (2015), Tran et al. (2018), Warren et al. (2018), Li et al. (2015), Ham et al. (2019), Milton et al. (2023)

### 5.1 Enzymatic degradation of inhibitory chondroitin sulfate

One of the most extensively investigated therapeutic strategies for promoting neural repair involves the enzymatic degradation of inhibitory CS chains within the ECM (Bradbury et al., 2002; Moon et al., 2001; Massey et al., 2006). The bacterial enzyme chondroitinase ABC (ChABC), originally isolated from *Proteus vulgaris*, cleaves CS chains and thereby disrupts the inhibitory matrix that accumulates after CNS injury. Numerous experimental studies have demonstrated that ChABC treatment enhances axonal regeneration, promotes synaptic plasticity (Gogolla et al., 2009; Pizzorusso et al., 2002), and improves functional recovery in animal models of spinal cord injury (García-Alías et al., 2009).

Mechanistically, digestion of CS chains diminishes the ability of CSPGs to engage inhibitory receptors such as PTPσ, thereby relieving growth cone inhibition. In addition, removal of CS chains may expose underlying extracellular matrix components that provide permissive substrates for axonal extension. Consistent with these mechanisms, ChABC treatment has been shown not only to facilitate regeneration of injured axons but also to promote sprouting of spared spinal pathways after injury (Massey et al., 2006). Such compensatory axonal growth contributes to the reorganization of residual neural circuits and is thought to underlie the functional improvements observed in experimental models.

Beyond its effects on axonal growth, enzymatic digestion of CS chains has also been shown to enhance synaptic plasticity in the adult CNS (Gogolla et al., 2009; Pizzorusso et al., 2002). Notably, degradation of perineuronal nets by ChABC reactivates ocular dominance plasticity in the adult visual cortex, indicating that CSPG-rich extracellular matrices function as key regulators that restrict structural plasticity in the mature nervous system (Pizzorusso et al., 2002).

Despite the promising effects observed in experimental studies, several challenges remain for the clinical translation of ChABC-based therapies. These include issues related to enzyme stability, delivery to the injury site, and potential immune responses. Nevertheless, ongoing efforts to develop stabilized enzyme variants and gene therapy–based delivery strategies continue to advance this approach toward potential clinical applications.

### 5.2 Synthetic and biomimetic GAGs

Another emerging strategy involves the development of synthetic or biomimetic GAG molecules designed to modulate extracellular signaling pathways that regulate axonal growth. Advances in chemical glycobiology have enabled the generation of defined GAG oligosaccharides with precisely controlled sulfation patterns, allowing researchers to investigate how specific structural motifs influence receptor binding and downstream signaling.

Synthetic GAG mimetics can function through several mechanisms. Some molecules are designed to competitively bind to GAG-recognition domains on neuronal receptors, thereby blocking inhibitory interactions between CSPGs and their receptors such as PTPσ. Other approaches aim to reproduce the growth-promoting functions of heparan sulfate by facilitating the interaction of growth factors with their cognate receptors, thereby enhancing pro-regenerative signaling pathways.

Pharmacological targeting of CSPG receptor signaling has also been explored as a strategy to promote axonal regeneration. For example, the low–molecular weight heparin derivative enoxaparin has been reported to antagonize PTPσ and enhance functional recovery after spinal cord injury. In experimental spinal cord injury models, administration of enoxaparin attenuated CSPG-mediated inhibitory signaling, resulting in enhanced axonal sprouting and improved locomotor recovery (Ito et al., 2021). These findings support the concept that small molecules capable of disrupting CSPG–PTPσ interactions may represent a promising therapeutic approach for promoting neural repair after central nervous system injury.

Biomaterial-based strategies have also been developed to recreate permissive extracellular environments for axonal growth. For instance, engineered electrospun scaffolds incorporating cellulose sulfate, a GAG-mimetic polymer, have been shown to enhance neurite extension and increase nerve growth factor binding compared with scaffolds containing native chondroitin sulfate (Menezes et al., 2019). More broadly, biomaterials incorporating defined GAG structures can function as artificial extracellular matrices that guide axonal growth and support neural tissue repair. Collectively, these studies suggest that artificial polymers mimicking sulfated GAGs can modulate axonal growth by recreating growth-permissive extracellular microenvironments.

### 5.3 Modulation or blocking of GAG sulfation patterns

Given that sulfation motifs play critical roles in determining GAG function, another therapeutic strategy involves manipulating sulfation patterns within GAG chains. The biosynthesis of sulfated GAGs is regulated by a variety of sulfotransferases and epimerases that act during polysaccharide assembly in the Golgi apparatus. Altering the expression or activity of these enzymes can modify the structural properties of GAG chains and thereby influence their biological functions.

Experimental studies have shown that changes in specific sulfation patterns can significantly affect axonal responses to extracellular matrix molecules. For example, a specific antibody against CS-E was shown to restore axonal regeneration after injury (Brown et al., 2012). In addition, protamine, a clinically used cationic peptide known for its strong affinity for sulfated GAGs, has been reported to neutralize the inhibitory activity of CS. In a mouse model of spinal cord injury, systemic administration of protamine alleviated CSPG-mediated inhibition, resulting in enhanced axonal regeneration across the lesion site and improved locomotor recovery (Ozaki et al., 2025). These findings suggest that pharmacological masking of sulfated GAGs chains represents a potential therapeutic strategy for promoting axonal regeneration after central nervous system injury These findings suggest that targeted manipulation or blocking of GAG sulfation patterns could provide a means of reshaping the extracellular environment to favor neural regeneration.

Although this strategy remains at an early stage, advances in genome editing and RNA-based technologies may facilitate the precise modulation of GAG-modifying enzymes in neural tissues. Such approaches could enable the selective reprogramming of extracellular matrix composition in injured or diseased regions of the CNS.

### 5.4 Genetic targeting of GAG biosynthetic pathways

A complementary approach involves genetic manipulation of enzymes involved in GAG biosynthesis. The assembly of GAGs chains requires a coordinated set of glycosyltransferases, sulfotransferases, and epimerases that collectively determine the structure of the resulting polysaccharides. Disruption or modification of these biosynthetic enzymes can alter the composition and functional properties of the extracellular matrix.

Studies using genetic models have demonstrated that changes in GAG biosynthesis can significantly influence neural development, axon guidance, and regeneration. Genetic manipulation of GAGs biosynthesis has also been explored as a strategy to promote axonal regeneration after central nervous system injury. For example, deletion of Chondroitin sulfate *N*-acetylgalactosaminyltransferase-1 (CSGalNAcT1), a key enzyme required for the initiation of CS chain biosynthesis, significantly improves functional recovery following spinal cord injury. In mice lacking this enzyme, CS synthesis is reduced, resulting in smaller glial scars and a less inhibitory extracellular environment (Takeuchi et al., 2013). Notably, this genetic manipulation also induces the upregulation of HS biosynthetic enzymes in neurons, leading to increased HS production, which is known to promote axonal growth. Consequently, simultaneous reduction of inhibitory CS and enhancement of growth-promoting HS creates a more permissive environment for axonal regeneration, resulting in greater recovery compared with wild-type mice and even with chondroitinase ABC treatment (Takeuchi et al., 2013). Knockdown of chondroitin sulfate synthase-1 (Chsy1), an enzyme required for chondroitin sulfate chain elongation, reduces CS accumulation and suppresses versican deposition in the extracellular matrix, thereby promoting axonal regeneration after peripheral nerve injury (Liu et al., 2023). Regarding to KS, as mentioned above, genetic deletion of GlcNAc6ST1, a key enzyme required for KS biosynthesis, results in the loss of KS expression and enhances neurite outgrowth and functional recovery after CNS injury, indicating that KS acts as an inhibitory component of the ECM (Ito et al., 2010; Zhang et al., 2006). These findings highlight the potential of targeting GAG biosynthetic pathways as a strategy to modulate neuronal environments.

Future therapeutic approaches may combine genetic manipulation with biomaterial or pharmacological strategies to achieve precise control over ECM composition. Such combinatorial approaches could provide powerful tools for promoting neural repair in conditions such as spinal cord injury, traumatic brain injury, and neurodegenerative diseases.

### 5.5 Targeting of GAG receptors

In addition to enzymatic degradation of inhibitory extracellular matrix components, direct modulation of receptor-mediated signaling has emerged as another strategy to overcome axon growth inhibition in the injured CNS. In particular, as discussed above, receptor-type protein tyrosine phosphatases, including PTPσ, have been identified as key receptors that mediate the inhibitory effects of CSPGs. Activation of these receptors by CS chains triggers intracellular signaling pathways that suppress growth cone motility and limit axonal extension. Therefore, strategies that interfere with RPTP signaling have attracted considerable attention as potential therapeutic approaches for promoting neural repair.

Though specific low molecular weight inhibitors for RPTP are rarely available, one such strategy involves the use of wedge-domain peptides derived from receptor-type protein tyrosine phosphatases (Xie et al., 2006). The wedge domain is a conserved structural element located near the catalytic domain of RPTPs and is thought to regulate phosphatase activity through conformational interactions that influence receptor dimerization. Peptides derived from this domain have been shown to inhibit phosphatase activity by disrupting these regulatory interactions, thereby modulating downstream signaling pathways. Experimental studies have demonstrated that wedge peptides can suppress the inhibitory signaling mediated by CSPG receptors, leading to enhanced neurite outgrowth *in vitro* and improved axonal growth in experimental models (Gardner et al., 2015; Lang et al., 2015; Tran et al., 2018; Warren et al., 2018). Mechanistically, inhibition of RPTP phosphatase activity is thought to maintain higher levels of tyrosine phosphorylation in intracellular signaling proteins that regulate cytoskeletal dynamics and growth cone behavior, including cortactin. By preventing excessive dephosphorylation events, wedge peptides may preserve pro-growth signaling pathways that support axonal extension. These findings support the concept that targeting intracellular signaling downstream of CSPG receptors represents a viable strategy for promoting neural regeneration. One notable example is NVG-291, a synthetic peptide derived from the regulatory wedge domain of PTPσ from NervGen Pharma. Preclinical studies have demonstrated that NVG-291 promotes axonal sprouting, synaptic plasticity, and functional recovery in models of nerve injury (Gardner et al., 2015; Lang et al., 2015; Tran et al., 2018; Warren et al., 2018; Li et al., 2015; Ham et al., 2019; Milton et al., 2023). Based on these findings, early-phase clinical trials have been initiated to evaluate the safety and therapeutic potential of this compound in humans. Phase 1 studies in healthy volunteers reported acceptable safety and pharmacokinetic profiles, enabling subsequent trials in patients with spinal cord injury. Ongoing Phase 1b/2a clinical studies are currently investigating the effects of NVG-291 in individuals with cervical spinal cord injury, with preliminary observations suggesting improvements in motor function and electrophysiological measures of neural connectivity. Although these results remain preliminary, they highlight the therapeutic potential of targeting GAG receptor signaling pathways for neural repair.

## 6 Future perspectives and emerging concepts

Despite substantial progress in understanding the roles of GAGs in neural biology, many fundamental aspects of GAG-mediated regulation of axonal behavior remain incompletely understood. Over the past decade, advances in glycobiology and neuroscience have revealed that GAGs are not merely structural components of the extracellular matrix but dynamic regulators of cellular signaling. Nevertheless, deciphering the precise molecular mechanisms through which diverse GAG structures influence neuronal responses remains a major challenge.

One key issue concerns the structural complexity of GAG chains. Unlike proteins or nucleic acids, GAGs are not directly encoded by a template-driven genetic mechanism. Instead, their structures are generated through coordinated activities of multiple biosynthetic enzymes that introduce sulfation, epimerization, and chain elongation modifications during polysaccharide assembly. This biosynthetic process produces highly heterogeneous GAG populations, making it difficult to define the precise structural motifs responsible for specific biological activities. Consequently, the identification of functionally relevant sulfation motifs and domain structures remains an important area of investigation.

Recent technological advances are beginning to address these challenges. High-resolution mass spectrometry, glycomic profiling, and emerging glycoproteomic approaches are providing unprecedented insights into the structural diversity of GAG chains in biological tissues. These analytical platforms allow researchers to characterize sulfation patterns, domain organization, and chain heterogeneity at increasing levels of detail. Applying such technologies to neural tissues may reveal how specific GAG structures are dynamically regulated during development, injury, and repair.

Another important direction involves understanding how multiple extracellular signaling systems interact within the neural microenvironment. Axonal responses to extracellular cues are governed by a complex network of signaling pathways that integrate inputs from proteoglycans, growth factors, cell adhesion molecules, and myelin-associated inhibitors. GAG chains occupy a central position within this network because they can simultaneously interact with numerous ligands and receptors. Future studies will therefore need to examine GAG signaling not in isolation but as part of a broader extracellular signaling landscape.

From a therapeutic perspective, targeting GAG-mediated pathways represents an attractive strategy for promoting neural repair. Experimental approaches such as enzymatic digestion of inhibitory chondroitin sulfate chains, development of synthetic GAG mimetics, and manipulation of sulfation pathways have demonstrated promising results in preclinical models. However, translating these strategies into clinical therapies will require improved methods for controlling GAG structures and delivering interventions to specific regions of the nervous system. Advances in gene editing, biomaterials, and drug delivery technologies may provide new opportunities to overcome these challenges.

An emerging conceptual framework is the idea that GAGs function through a molecular “GAG code” that regulates neuronal behavior. According to this hypothesis, specific combinations of sulfation motifs, domain structures, and chain lengths encode biochemical signals that determine whether the extracellular environment promotes or restricts axonal growth. Changes in this structural code may therefore influence neuronal plasticity, regeneration, and circuit remodeling.

Deciphering this potential GAG code represents a major Frontier in both glycobiology and neurobiology. A deeper understanding of how structural variations in GAGs control receptor signaling and cellular responses could reveal new principles governing extracellular regulation of neural function. Ultimately, integrating structural glycomics with functional neuroscience may enable the rational design of strategies to manipulate extracellular matrix signals and promote regeneration in the injured or diseased nervous system.
